# Mesenchymal Stem Cell-Based Therapy for Stroke: Current Understanding and Challenges

**DOI:** 10.3389/fncel.2021.628940

**Published:** 2021-02-09

**Authors:** Weifeng Li, Linli Shi, Bei Hu, Yimei Hong, Hao Zhang, Xin Li, Yuelin Zhang

**Affiliations:** ^1^Department of Emergency Medicine, Guangdong Provincial People’s Hospital, Guangdong Academy of Medical Sciences, Guangzhou, China; ^2^The Second School of Clinical Medicine, Southern Medical University, Guangzhou, China; ^3^Faculty of Pharmacy, Bengbu Medical College, Bengbu, China

**Keywords:** mesenchymal stem cell, stroke, cell therapy, mechanisms, challenges

## Abstract

Stroke, the most prevalent cerebrovascular disease, causes serious loss of neurological function and is the leading cause of morbidity and mortality worldwide. Despite advances in pharmacological and surgical therapy, treatment for functional rehabilitation following stroke is limited with a consequent serious impact on quality of life. Over the past decades, mesenchymal stem cell (MSCs)-based therapy has emerged as a novel strategy for various diseases including stroke due to their unique properties that include easy isolation, multipotent differentiation potential and strong paracrine capacity. Although MSCs have shown promising results in the treatment of stroke, there remain many challenges to overcome prior to their therapeutic application. In this review, we focus on the following issues: the scientific data from preclinical studies and clinical trials of MSCs in the treatment of stroke; the potential mechanisms underlying MSC-based therapy for stroke; the challenges related to the timing and delivery of MSCs and MSC senescence.

## Introduction

Stroke, one of the major diseases of the central nervous system, is a global health problem with limited treatment options. It is classified as hemorrhagic (13%), caused by rupture of blood vessels, or ischemic (87%), caused by disruption of blood supply (Kalladka and Muir, 2014). With an increasing elderly population, the mortality and morbidity of stroke are increasing. Approximately 15 million individuals worldwide are affected by stroke each year, of whom 5 million will ultimately die and 5 million will suffer long-term disability (Roy-O’Reilly and McCullough, 2014). Ischemic stroke is caused by occlusion of a supply artery due to embolus or thrombus. As a result of cerebral ischemia, excitatory amino acids react with tissues and generate a large number of calcium ions and free radicals. This produces carbon monoxide with consequent irreversible necrosis of brain cells (Rastogi et al., 2006). The necrotic portion, also known as the ischemic core, is surrounded by the peri-infarct region or penumbra that represents the functionally impaired but potentially salvageable tissue and is the primary target for the developing neuroprotective strategies (Candelario-Jalil and Paul, 2021). Rapid restoration of cerebral blood flow is the focus of the treatment for acute stroke. Currently, there are no proven options for stroke patients aside from dissolution of thrombus via tissue plasminogen activator (e.g., alteplase), or mechanical thrombectomy (Hacke et al., 2008; Powers et al., 2015; Saver et al., 2016). However, thrombolysis has a narrow therapeutic window, being clinically effective only within 4.5 h after stroke and losing its effect when the thrombus is large or the stroke is extensive (Bhaskar et al., 2018). Fewer than 5% of ischemic stroke patients receive such treatment and still suffer post-treatment neurological deficits with no therapy available to promote recovery (Lyden et al., 2019). Mechanical thrombectomy exhibits the significant therapeutic efficacy in acute ischemic stroke caused by intracranial proximal artery occlusion. However, this technique is not yet fully developed, and the efficacy and safety of endovascular reperfusion beyond 6 h remains controversial (Berkhemer et al., 2015; Smith, 2019). Limited numbers of stroke patients can benefit from these approaches and achieve good outcomes (Detante et al., 2017).

Over the past decades, stem cell-based therapy has attracted great interest as an emerging treatment in stroke in the hope that it can repair the damaged central neural networks (Wan Safwani et al., 2017; Choi et al., 2018). Stem cell therapy displays significant effects of functional improvement for ischemic stroke, offering hope for the preservation of neural tissue in the acute phase of stroke and the replacement of lost tissue in the chronic stage (Wei et al., 2017).

MSCs are pluripotent, non-hematopoietic stem cells with the ability to differentiate into a diverse number of cell lineages, including chondrocytes, osteoblasts, and neuron-like cells (Uccelli et al., 2006; Williams and Keating, 2008). They can be isolated from almost all tissues in mammals including bone marrow (BM), adipose tissue or other tissues (Pinho et al., 2020) and are easy to culture and effectively expand. BM-MSCs are the most common, while in recent years adipose-derived MSCs have become increasingly popular due to their easy availability and high yield (Faghih et al., 2017; Perteghella et al., 2017). MSCs from bone marrow, adipose tissue have full trilineage (adipogenic, osteogenic, and chondrogenic) differentiation capacity and excellent immunomodulatory properties compared to other sources, and therefore represent the optimal stem cell sources for tissue engineering and regenerative medicine (Heo et al., 2016). Even in the acute stage of stroke, MSCs are suitable for transplantation and have substantial neurotrophic effects (Walczak et al., 2008). In addition, MSCs derived from adult tissues pose no risk of tumorigenesis and their low expression of major histocompatibility complex (MHC)-I and MHC-II antigens eliminates the need for immunosuppression following allogeneic administration (Bhatia and Hare, 2005; Williams and Hare, 2011). The therapeutic effects of MSCs are mediated by many mechanisms including anti-inflammation, anti-apoptosis, angiogenesis, and neurogenesis. They have become the focus of many preclinical and clinical studies (Li et al., 2016; Moniche et al., 2016). This review will focus on the application of MSCs in the treatment of stroke.

## Preclinical Studies

The application of MSCs in the treatment of stroke has been studied for nearly two decades. Several recent animal studies are summarized in Table 1. In most studies, Sprague Dawley (SD) rats or Wistar rats were used to establish a model of cerebral ischemia, induced by middle cerebral artery occlusion (MCAO). It has been shown that transplantation of MSCs following ischemic stroke promotes improvement of cerebral function (Toyoshima et al., 2015; Moisan et al., 2016; Hu et al., 2019) effectively protects ischemic neurons and restores brain damage (Son et al., 2019). However, several studies used young adult and healthy animals, without taking into account the fact that a large proportion of ischemic stroke patients are elderly and the presence of comorbidities such as hypertension and diabetes (Howells et al., 2010; Laso-Garcia et al., 2019). Herein, these animal models create the barriers to the translation of the findings to clinical trials. Therefore, we here focused on studies that incorporated comorbidities into animal models of stroke.

**TABLE 1:** Overview of animal studies of MSC-based therapy for stroke.

**Animal species**	**Stroke type**	**Comorbidity**	**Cell source**	**Cell number**	**Delivery route**	**Timing**	**Results**	**References**
SD	MCAO	–	BM	1 × 10^5^	IA (carotid artery)	10 days	Neuronal regeneration	Hu et al., 2019
SD	MCAO	–	BM	3 × 10^6^	IV (tail vein)	8 days	Angiogenesis	Moisan et al., 2016
Wistar	MCAO	–	BM	1 × 10^6^	IA	1, 6, 24, and 48 h	Reduce infarction volume	Toyoshima et al., 2015
SD	MCAO	–	BM	2 × 10^5^	IC (brain tissue)	1 days	Protect ischemic neurons	Son et al., 2019
Wistar	MCAO	Aging	BM	2 × 10^6^	IA (carotid artery)	1 days	Long-term improvement in functional outcome	Shen et al., 2007a
SD	MCAO	Aging	BM	1 × 10^5^	IA	6 h	Improve the functional outcome	Saraf et al., 2019
SHR	Stroke prone	Hypertension	BM	1 × 10^6^	IC (atlanto-occipital membrane)	–	Neuroprotective and antioxidant potential	Calio et al., 2014
SHR	MCAO	Hypertension	Placenta	1 × 10^6^	IV (tail vein)	8 and 24 h	Functional recovery	Kranz et al., 2010
SD	MCAO	Hyperglycemia	Adipose tissue	1 × 10^6^	IV (tail vein)	48 h	Neurological recovery	Gomez-de Frutos et al., 2019
Wistar	MCAO	Diabetes	BM	5 × 10^6^	IV (tail vein)	24 h	Neurorepair effects	Cui et al., 2016
Wistar	MCAO	Diabetes	BM	3 × 10^11^	IV (tail vein)	3 days	Improve the functional outcome	Venkat et al., 2020

*MCAO, middle cerebral artery occlusion; BM, bone marrow; IA, intra-arterial; IC, intracerebral; IV, intravenous.*

It has been estimated that about 75% of strokes occur in the elderly (Yousufuddin and Young, 2019). Shen et al. (2007a) selected 10–12 month-old female retired breeder rats to establish an ischemic stroke model, and confirmed the long-term neurological protective effects of MSC on ischemic stroke. In addition, Saraf et al. demonstrated that stroke induced CaN hyperactivation, triggering an apoptotic pathway in neurons that further led to neuronal death in middle-aged ovariectomized female rats. MSCs treatment rescued neurons and promoted neuronal survival via reducing CaN expression (Saraf et al., 2019). Hypertension is the major risk factor for all types of stroke (Hong, 2017; Cipolla et al., 2018), Hypertensive ischemic stroke models mostly use stroke-prone spontaneously hypertensive rats (SHRSP), an animal model that develops 100% hypertension without genetic modification, has cerebrovascular pathology and physiology very similar to that of human hypertension, and induces spontaneous strokes at a rate of more than 60% (Liao et al., 2013). Calio et al. (2014) demonstrated that MSCs induced an increase in the anti-apoptotic gene Bcl-2 and protected brain tissue through anti-apoptosis and antioxidation, suggesting that MSCs have a protective effect on neuronal cells in SHRSP rats. In another study, placental derived MSCs treatment greatly improved functional recovery and reduced infarct size in mice with hypertensive ischemic (Kranz et al., 2010). Diabetes is a definite risk factor for stroke. Patients with diabetes have an increased probability of developing ischemic stroke, and hyperglycemia exacerbates microvascular and macrovascular damage in ischemic strokes (Rehni et al., 2017; Lau et al., 2019). Therefore, studying diabetic stroke models is of great importance. It has been reported that 6 weeks after permanent MCAO, lesions were more severe in the hyperglycemic group than in the non-hyperglycemic group. Although human adipose tissue-derived MSCs treatment for hyperglycemic stroke rats did not reduce lesion size, it significantly improved neurological function (Gomez-de Frutos et al., 2019). Cui et al. (2016) demonstrated the beneficial effects of BM- MSCs in type 1 diabetic rats with stroke via mediating miR-145. In type 2 diabetic Wistar rats with stroke, treatment with exosomes harvested from BM-MSCs significantly improved blood-brain barrier (BBB) integrity, increased white matter remodeling, and promoted neural repair (Venkat et al., 2020).

Comorbidities in humans can profoundly affect stroke pathophysiology, lesion development, and recovery (Cho and Yang, 2018). Despite of the beneficial effects of MSCs, more preclinical studies are warranted to exploring MSC therapy for stroke due to the limited number of relevant studies in stroke comorbidity models. Therefore, the importance of using preclinical comorbidity model should be emphasized when establishing guidelines on how to improve the validity of animal models of stroke.

## Clinical Trials

Although preclinical studies have shown that MSCs displays beneficial effects on stroke (Moisan et al., 2016; Cunningham et al., 2018), the safety problems inflammation, tumor development, metastasis in clinical trials have been reported (Gazdic et al., 2015; Dhere et al., 2016; Volarevic et al., 2018). Over the past few decades, the safety, feasibility and effectiveness of MSCs in the treatment of stroke have been widely studied in clinical trials (Table 2). Previous clinical trials have shown that MSCs isolated from different tissues have shown the high efficiency for stroke treatment (Detante et al., 2017; Wechsler et al., 2018; Chrostek et al., 2019; Cui et al., 2019; Suda et al., 2020). Several routes of delivery have been proposed including intracerebral (IC), intra-arterial (IA), and intravenous (IV) (Toyoshima et al., 2017). Among them, the intracerebral pathway is the most effective and invasive route. In contrast, the intravenous pathway is the least invasive, but the number of MSC cells reaching the injured brain is the most limited. The intra-arterial pathway is relatively neutral. In 2005, autologous BMSCs transplantation was performed intravenously for the first time in five patients with acute ischemic stroke and no adverse reactions were reported (Bang et al., 2005). In 2010, a large long-term study evaluated the safety and efficacy of autologous intravenous BMSCs transplantation and got similar results (Lee et al., 2010). In 2011, the reduction in infarct lesion volume and recovery of neurological function were obtained following administration of serum-expanded autologous BMSCs to chronic stroke patients (Honmou et al., 2011). Afterwards, a phase I/II study of intracerebral cell transplantation in patients with chronic stroke has reported that intracerebral transplantation of genetically modified MSCs significantly improved neurological function (Steinberg et al., 2016, 2018). A single-center, open-label Randomized Controlled Trial study showed that intravenous injection of autologous BMSCs also improved the motor function, suggesting that MSCs treatment is feasible therapeutic strategy for stroke (Jaillard et al., 2020).

**TABLE 2:** Clinical trials of MSC-based therapy for stroke.

**Phase**	**Patients number**	**Delivery route**	**Cell source**	**Cell number**	**Timing**	**Results**	**References**
I	5	IV	Autologous BM-MSCs	1 × 10^8^	7 days	Improve in BI	Bang et al., 2005
II	16	IV	Autologous BM-MSCs	5 × 10^7^	5–7 weeks	Improve in mRS	Lee et al., 2010
I	12	IV	Autologous BM-MSCs	1 × 10^8^	36–133 days	Improve in NIHSS	Honmou et al., 2011
I	8	IV	Autologous BM-MSCs	5–6 × 10^7^	3 months–1 year	Improve in Fugle-Meyer and mRS, increase in number of cluster activation of Brodmann areas BA 4 and BA 6	Bhasin et al., 2011
II	20	IV	Allogeneic AD-MSCs	1 × 10^6^ cells/kg	2 week	Safe and effective	Diez-Tejedor et al., 2014
I/IIa	18	IC	Modified MSCs (SB623)	dose-escalation: 2.5 × 10^6^, 5.0 × 10^6^, or 10 × 10^6^	6–60 months	improve in ESS, NIHSS, Fugle-Meyer	Steinberg et al., 2016, 2018
II	48	IA	BM-ALDH^*br*^cells	0.5 × 10^5^–2.5 × 10^7^	9–15 days	Safe	Savitz et al., 2019
I	10	IV	Allogeneic UC-MSCs	5 × 10^6^–5 × 10^7^/Kg	7–10 days	Safe and feasible	Laskowitz et al., 2018
II	16	IV	Autologous BM-MSCs	10 × 10^7^–30 × 10^7^	14 days	Improve in motor-NIHSS,Fugle-Meyer, task-related fMRI activity	Jaillard et al., 2020

*BI, Barthel Index; mRS, modified Rankin Scale; NIHSS, National Institute of Health Stroke Scale; ESS, European Stroke Scale; BM, bone marrow; MSC, mesenchymal stem cell; UC-MSC, umbilical cord mesenchymal stem cell; ALDH^*br*^, aldehyde dehydrogenase bright; IA, intra-arterial; IC, intracerebral; IV, intravenous.*

Recently, Savitz et al. (2019) conducted a randomized, sham controlled, phase II trial in which autologous BM-derived aldehyde dehydrogenase (ALDH)-bright cells were transplanted intra-arterially to patients with disabling middle cerebral artery stroke and showed no adverse events in the treatment patients group, although there was a higher incidence of small lesions on MRI. In addition, superparamagnetic iron oxide-labeled BMSCs were also used to treat stroke patients and proved to be safe and tolerated (Shichinohe et al., 2017). Compared to BMSCs, adipose tissue-derived mesenchymal stem cells (AD-MSCs) are easier and safer to prepare without adverse side effects and ethical concerns (Ra et al., 2011; Gutierrez-Fernandez et al., 2013a,b). In 2014, allogeneic intravenous AD-MSCs transplantation was carried out in patients with subacute stroke. The results demonstrated that allogeneic AD-MSCs had no association with the development of tumors. The study concluded that AD-MSCs-based cell therapy was safe and could promote rehabilitation of stroke (Diez-Tejedor et al., 2014).

Human umbilical cord-derived MSCs (hUC-MSCs) have great advantages for stroke treatment due to low immunogenicity and no substantial ethical problems (Yin et al., 2019). A Phase I clinical trial using hUC-MSCs for acute stroke treatment demonstrated a significant functional recovery, indicating that hUC-MSCs treatment is safe and feasible option for acute stroke (Laskowitz et al., 2018).

Although MSC transplantation has been proven to be safe and feasible in small phase I/II trials, no significant improvement was observed in a randomized controlled intravenous phase II trial (Hess et al., 2017). Their efficacy in the treatment of ischemic stroke therefore remains controversial (Hess et al., 2017). Several reasons are proposed for the inconsistent results of MSC transplantation in ischemic stroke. First, trials differ in patient numbers, dose and type of cell delivery, timing of cell therapy, and treatment modalities. Second, the location/extent and severity of the lesions are different. The studies also applied different evaluation criteria when assessing neurological function and adverse reactions. More optimized and well-designed large sample multicenter studies are needed to explore the therapeutic efficacy of MSCs in ischemic stroke.

## Potential Mechanisms of MSC Therapy for Stroke

**FIGURE 1:**
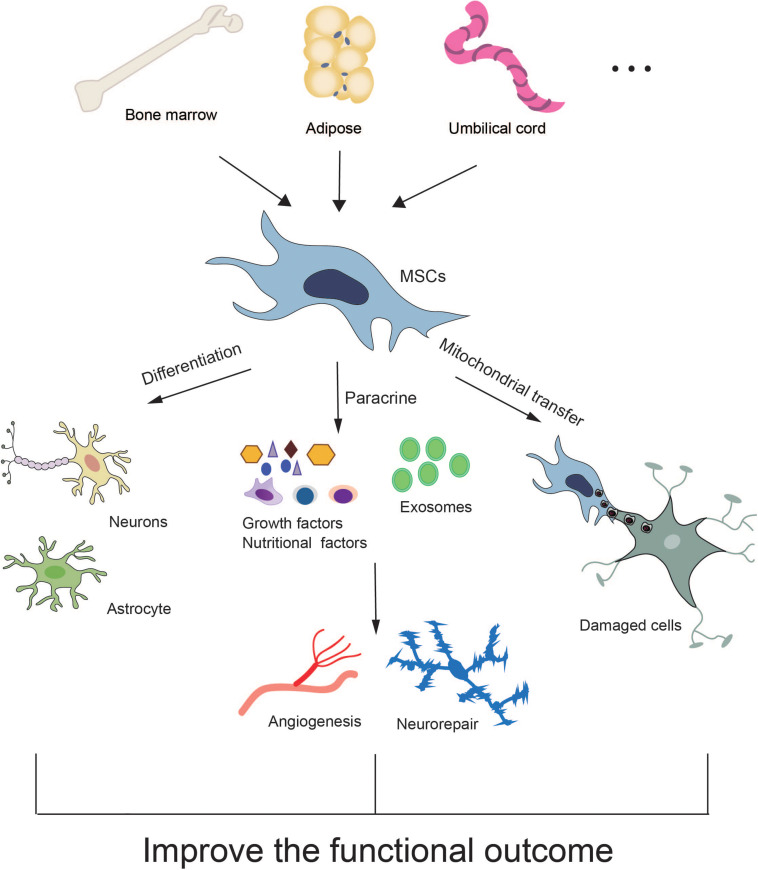
Potential mechanisms of MSC therapy for stroke.

Currently, the underlying mechanisms of MSC-based therapy for stroke have not been fully elucidated. Many experimental studies have revealed that MSCs protect against stroke through multiple mechanisms including direct differentiation, paracrine effects and mitochondrial transfer (Figure 1).

### MSC Differentiation

MSCs are pluripotent adult mesenchymal cells with the ability of self-renewal and multi-differentiation (Klimczak and Kozlowska, 2016). In the presence of injury and inflammation, MSCs are directly transplanted or homed to the damaged site (Shi et al., 2012). In a specific microenvironment of a tissue or organ, MSCs have the ability to divide and proliferate, differentiate and develop into the same cell type as the tissue or organ, including neuronal cells, and effect repair (Clevers et al., 2014; Shi et al., 2016). It has been reported that MSCs isolated from human umbilical cord can differentiate into neuron-like cells and maintain their immunomodulatory and antioxidant activities (Li et al., 2012).

### Paracrine Effects

There is accumulating evidence that the ability of MSCs to differentiate into neuron cells cannot on its own explain the predominant benefits of MSC-based therapy (Ghittoni et al., 2005; Wang et al., 2012; Bang and Kim, 2019; Brown et al., 2020). Compared with BM-MSC transplantation, infusion of MSC-derived conditioned medium has been shown to equally improve injured brain function (Ghittoni et al., 2005; Gnecchi et al., 2006; Volarevic et al., 2011). A paracrine effect of MSCs is thus concluded to be a major mechanism underlying the benefits of MSC-based therapy for stroke (Liang et al., 2014). There is a close interaction between the soluble factors derived from MSCs and immune cells (such as dendritic cells, lymphocytes, natural killer cells, and macrophages) (Gao et al., 2014). A paracrine role is reflected in immune regulation: MSCs secrete soluble factors through direct cell-cell interaction, are involved in immune regulation and induce immune tolerance, and can improve and regulate the destructive inflammatory response (Wu et al., 2017; de Witte et al., 2018). Numerous paracrine components form a complex exocrine factor network to ensure the stability of cells and enhance the regeneration response. Many MSC-based tissue repair models are largely dependent on the paracrine action of MSCs (Weiss and Dahlke, 2019; Wu et al., 2020). Another manifestation of their paracrine effect is in the promotion of angiogenesis (De Luca et al., 2011; Gnecchi et al., 2016; Chen et al., 2020). Both basic fibroblast growth factor and vascular endothelial growth factor induce endothelial cell proliferation and migration to form new vascular branches from existing vascular branches (Gnecchi et al., 2006). The various nutritional factors secreted by MSCs, including enzymes, growth factors, chemokines, matrix metalloproteinases, and adhesion molecules, all have an effect on several key steps of angiogenesis. They can induce the proliferation, migration and tubular formation of vascular endothelial cells, as well as inhibit apoptosis of endothelial cells (Kinnaird et al., 2004; Rehman et al., 2004). Transplantation of adipose tissue-derived MSCs has also been shown to promote angiogenesis and improve behavioral recovery in SD rats after MCAO operation (Mu et al., 2019). BM-MSCs can increase the expression of astrocyte-derived VEGF and BDNF in the ischemic boundary zone after stroke and promote angiogenesis, as well as the recruitment and proliferation of reactive astrocytes, leading to nerve injury repair (Guo et al., 2012; Zhang et al., 2017). Moreover, Human BM-MSCs have been shown to increase cerebral vascular generation in stroke lesions by releasing endogenous angiogenic factors that enhance the stability of new blood vessels (Moisan et al., 2016). Therefore, the paracrine effect of stem cells is likely to play an important role in increasing capillary density and angiogenesis in the damaged brain.

Notably, exosomes are the most important agents in the process of information transmission and inducing repair for many secreted cytokines (Elahi et al., 2020). The paracrine effect produced by their external secretion plays a critical role in stroke recovery (Xin et al., 2013a; Hao et al., 2014; Zhang Y. et al., 2015; Zhang et al., 2019). Exosomes from MSCs are 30–100 nm diameter lipid particles with a double membrane structure containing micro RNAs, mRNAs, DNAs, and bioactive substances such as protein and lipids. They display similar properties and functions to MSCs including low immunogenicity and the ability to stimulate nerve vascular repair with no risk of tumor formation (Yaghoubi et al., 2019). Increasing lines of evidence have confirmed that exosomes contribute significantly to the benefits of cell-based therapies, including the treatment of stroke, traumatic brain injury and other neurological diseases (Xin et al., 2013b; Zhang Y. et al., 2015; Stonesifer et al., 2017; Zhang et al., 2019). Dabrowska et al. (2019) demonstrated that intra-arterial delivery of exosomes derived from BM-MSCs reduced neuroinflammation induced by focal brain injury in ischemic stroke and this effect was comparable with that of transplanted BM-MSCs. In some mouse models of stroke, the long-term neuroprotection afforded by MSCs was closely associated with enhanced angiogenesis, and reduced post-ischemic immunosuppression (i.e., B cells, natural killer cells, and T-cell lymphocytosis), providing an appropriate external environment for successful brain remodeling (Doeppner et al., 2015; Hu et al., 2016). The secretion of a wide range of bioactive molecules is now considered the major mechanism of MSC-based therapy.

### Mitochondrial Transfer

Mitochondrial transfer is a novel mechanism for stem cell therapy that has attracted wide attention. MSCs can transfer mitochondria to injured cells with mitochondrial dysfunction through a variety of ways to restore cell aerobic respiration and mitochondria function, leading to rescue of cell injury (Spees et al., 2006; Islam et al., 2012; Han et al., 2016). Mitochondrial dysfunction has been considered a sign of ischemia/reperfusion injury in the complex cell process, so mitochondrial transfer may be one of the mechanisms by which MSC treatment is of benefit for stroke (Han et al., 2020). Co-cultured BM-MSCs can transfer intact mitochondria through transient tunneling nanotubes (TNT) to damaged cells, restoring their mitochondrial function (Han et al., 2016). Yang et al. demonstrated that iPSC-MSCs could protect damaged PC12 cells by restoring mitochondrial function. This was not just due to the paracrine effect of MSCs, but also attributed to the mitochondria transferred from MSCs to the injured PC12 cells (Yang et al., 2020). There is accumulating evidence that mitochondrial transfer between MSCs and damaged cells is mainly mediated through tunneled nanotubes and microvesicles (Spees et al., 2006; Islam et al., 2012; Li et al., 2014; Murray and Krasnodembskaya, 2019). In addition, Babenko et al. (2015) reported that BM-MSCs can donate mitochondria to injured astrocytes and restore their mitochondrial function, demonstrating the protective function of MSCs on nerves. Tseng et al. (2020) demonstrated that transfer of mitochondria from MSCs to damaged neurons induced by oxidative stress *in vivo* and *in vitro* resulted in metabolic benefits. The researchers tagged BM-MSCs and tracked the transplanted mitochondria. They observed the mitochondria transfer and a protective effect on the damaged cerebral microvascular system in rats with cerebral ischemia (Liu et al., 2019; Yang et al., 2020). Thus, mitochondrial transfer from MSCs to damaged cells may offer a new avenue in the treatment of stroke.

## Challenges of MSC Therapy for Stroke

Although many animal studies and clinical trials of MSC-based therapy for stroke have obtained promising results, there remain many challenges to overcome before MSCs can be widely applied in clinical practice.

First, the optimum time for MSC administration remains controversial. Currently, most preclinical studies recommend transplanting MSCs during the acute stroke stage (<48 h). It has been reported that stroke can cause an increase in reactive oxygen species, activation of immune cells and production of pro-inflammatory cytokines in the acute phase, thus aggravating the secondary brain injury. MSC exosomes display immunomodulatory and neuroprotective effects, especially in the acute phase of stroke (Tobin et al., 2014; Vu et al., 2014). In contrast, some studies have shown that administration of MSCs in rats 1 month after stroke could also lead to neurological recovery (Shen et al., 2007b). MSCs can secrete many growth factors to activate the endogenous repair process, induce a decrease in glial scar and increase proliferation of cells in the subventricular area, promoting neurogenesis in the chronic phase of stroke (Shen et al., 2007b; Sinden et al., 2012). MSC transplantation has also been administered in the subacute or chronic stage of stroke in some clinical studies (Lalu et al., 2020). How to solve this knowledge gap and use available evidence to determine the optimal timing for cell therapy remains a major challenge.

Another challenge is to determine the best treatment. Although MSCs have shown general immune evasion and tolerance in clinical studies of stroke, an increasing number of preclinical studies have proved the therapeutic efficacy of conditioned medium (CM) and extracellular vesicles (EVs) derived from MSCs that reduces the dependence on and need for cells (Xin et al., 2013b; Cunningham et al., 2018). These cell-free substitutes can be cryopreserved with no concerns about cell survival. Cells can be preserved for a long time and transported around the world. Nevertheless, there is no clear consensus on the optimal culture conditions and pretreatment strategies to maximize the regenerative potential of MSC-derived CM or EVs (Reiner et al., 2017). Further clinical studies are needed to clarify their therapeutic value for stroke.

Third, the route of MSC administration is another major challenge. Previous studies have used both systemic and direct approaches such as IV, IA, and IC. Compared with more invasive routes (e.g., intrathecal and IC approaches), minimally invasive routes (e.g., IV and IA approaches) may cause less damage at the injection site although each route has its own advantages and disadvantages. How to choose a simpler and safer delivery route for MSCs is a major hurdle to their clinical application with much greater care required by the clinician.

Fourth, the best source of MSCs for stroke treatment has not been determined. Although most preclinical studies (>90%) use fresh MSCs from healthy, young donors, half of the clinical studies used autologous MSC products. Autologous MSCs may circumvent the logistical ethical problems and have been proven to be more effective than those obtained from healthy donors (Block et al., 2017; van de Vyver, 2017). Nonetheless, expanding enough stem cells for transplantation requires a long time, so it is impossible to use autologous MSC cells in the acute stage of stroke, especially from elderly patients or those with serious diseases. Genetic engineering or reprogramming to amplify MSCs can lead to uncontrolled proliferation and genetic abnormality, limiting their viability and therapeutic potential (Zhang J. et al., 2015). Furthermore, whether modified MSCs can successfully differentiate into fully functional neural cells in patients remains elusive (Strioga et al., 2012). One large randomized controlled clinical trial reported that cryopreserved allogeneic MSCs from healthy donors had poor viability and poor clinical efficacy (Matthay et al., 2019). Therefore, when using allogeneic cells from healthy donors care should be taken to assess their viability in order to match preclinical conditions.

Fifth, another challenge is the heterogeneity in study designs. The poor methodological rigorism of both preclinical and clinical studies may have contributed to the current conflicting results. Preclinical studies rarely adopted randomized or blinding designs, or carried out confirmatory studies, a pre-requisite of clinical trials (Macleod et al., 2008; Hirst et al., 2014). Similar problems also existed in clinical studies. Studies included RCT (Lee et al., 2010; Hess et al., 2017), single-arm trial (Steinberg et al., 2016), or case series (Honmou et al., 2011), and could not be compared. Previous clinical measures of efficacy reported included NIHSS, mRS, BI, Fugle-Meyer scale, and ESS. A unified method for evaluation of neurological function is lacking so it is difficult to reach a consistent conclusion about the safety and effectiveness of MSCs in clinical application. How to increase methodological rigorism is a great challenge for preclinical and clinical studies.

Sixth, senescence of MSCs has attracted extensive attention in recent years. The passage times of MSCs are limited. Extension of expansion time will inevitably lead to replicative senescence. Moreover, MSCs isolated from the elderly exhibit an aging phenotype with a decline in function, leading to decreased therapeutic efficacy (Wagner et al., 2008). Therefore, developing strategies to deal with MSC senescence is another future challenge (Kim and Park, 2017).

Finally,the comorbidities of patients are also the challenges for MSC therapy (Cui et al., 2019). Many stroke patients have comorbidities such as hypertension, diabetes and heart disease that may exert an impact on therapy efficacy (Chen et al., 2011). The medications such as antidiabetics and antiplatelet drugs often influence MSC function, limiting the therapeutic effects (Ortega et al., 2013). Unfortunately, most of preclinical studies haven’t investigated influence factors, leading to a big knowledge gap in translate stroke research to clinic.

## Conclusion

MSCs have many advantages: they are immune evasive, easy to harvest, expand and store for a long time, and convenient to manage in various ways. Additionally, their clinical use does not raise many ethical issues. Increasing evidence supports the potential of MSCs to treat stroke, but there are challenges to overcome. We have systematically reviewed the safety and efficacy of MSCs in the treatment of ischemic stroke and hemorrhagic stroke. In preclinical studies, MSC treatment has shown considerable efficacy in some neurological function tests, but there remains no large-scale randomized, double-blind, multicenter clinical study to prove their effectiveness. The heterogeneity of MSCs is the main barrier to their clinical application and therapeutic effect. Key parameters such as the source of MSCs, dosage, route of administration, administration time and other key parameters directly affect the application effect. More importantly, many clinical trials have similar limitations in detecting the role of MSCs, including small size, lack of a control arm, and inconsistent methods for use of MSCs. Homogenization and quality control are key issues in their clinical application. Future preclinical and clinical studies should consider adoption of a well-designed randomized controlled study design, method rigor and intervention measures, so as to determine the effect of MSC therapy in the treatment of stroke (Napoli and Borlongan, 2016; Squillaro et al., 2016). Nonetheless, despite these issues, MSCs have exciting potential as a means to protect neurons and improve the outcome for stroke patients.

## Author Contributions

WL and LS searched the literature and wrote the manuscript. BH, YH, and HZ searched the literature and provided comments. XL and YZ designed the study and wrote the manuscript. All authors contributed to the article and approved the submitted version.

## Conflict of Interest

The authors declare that the research was conducted in the absence of any commercial or financial relationships that could be construed as a potential conflict of interest.
